# Comprehensive Analysis of Microsatellite Instability in Canine Cancers: Implications for Comparative Oncology and Personalized Veterinary Medicine

**DOI:** 10.3390/ani14172484

**Published:** 2024-08-27

**Authors:** Eugenio Mazzone, Luca Aresu

**Affiliations:** Department of Veterinary Sciences, University of Turin, 10095 Grugliasco, Italy; luca.aresu@unito.it

**Keywords:** animal models, dogs, cancer, immunotherapy, microsatellite instability, high-throughput nucleotide sequencing

## Abstract

**Simple Summary:**

This research aims to address the significant knowledge gap regarding microsatellite instability (MSI) in canine cancers. While MSI has been extensively studied in human oncology, its prevalence and importance in canine tumors remain largely unexplored. We seek to provide a comprehensive analysis of MSI across various canine cancer types using a large dataset of whole-exome sequencing samples. By elucidating the landscape of MSI in canine cancers, the study aims to uncover potential implications for cancer development, progression, and treatment strategies. The findings from this research may significantly impact the veterinary oncology community by identifying new biomarkers for prognosis and treatment response, particularly in relation to immunotherapy approaches. Moreover, the study’s novel “MSI-burden” score and its correlation with tumor mutational burden could provide valuable insights into canine cancer biology. Ultimately, this research may open new avenues for targeted therapies and personalized medicine in veterinary oncology, potentially improving outcomes for canine cancer patients.

**Abstract:**

Microsatellite instability (MSI) is a crucial feature in cancer biology, yet its prevalence and significance in canine cancers remain largely unexplored. This study conducted a comprehensive analysis of MSI across 10 distinct canine cancer histotypes using whole-exome sequencing data from 692 tumor-normal sample pairs. MSI was detected in 64% of tumors, with prevalence varying significantly among cancer types. B-cell lymphomas exhibited the highest MSI burden, contrasting with human studies. A novel “MSI-burden” score was developed, correlating significantly with tumor mutational burden. MSI-high (MSI-H) tumors showed elevated somatic mutation counts compared to MSI-low and microsatellite stable tumors. The study identified 3632 recurrent MSI-affected genomic regions across cancer types. Notably, seven of the ten cancer types exhibited MSI-H tumors, with prevalence ranging from 1.5% in melanomas to 37% in B-cell lymphomas. These findings highlight the potential importance of MSI in canine cancer biology and suggest opportunities for targeted therapies, particularly immunotherapies. The high prevalence of MSI in canine cancers, especially in B-cell lymphomas, warrants further investigation into its mechanistic role and potential as a biomarker for prognosis and treatment response.

## 1. Introduction

Microsatellites (MS) are short, tandemly repeated nucleotide sequences dispersed across millions of loci within the genome. Their repetitive nature renders these sequences particularly susceptible to DNA polymerase slippage events during replication, resulting in variations in repeat length [1]. The mismatch repair (MMR) pathway identifies and corrects these errors, thereby safeguarding the genome against potentially deleterious mutations. When the MMR system is compromised, either through genetic mutations or epigenetic silencing, the frequency of spontaneous mutations in microsatellite regions increases, a phenomenon termed microsatellite instability (MSI) [2]. 

MSI serves as a molecular hallmark of MMR system defects and is associated with various malignancies. It is characterized by the spontaneous gain or loss of nucleotides within these repetitive tracts, indicative of MMR system disruption, leading to the accumulation of somatic mutations at high rates within microsatellites and the formation of novel microsatellite alleles. MSI has significant implications in oncology, particularly in the clinical management of human colorectal and endometrial cancers [3]. Traditionally, molecular investigations of MSI have employed PCR-based methods and immunohistochemistry (IHC) to assess the status of key MMR proteins [4]. However, recent advances in next-generation sequencing (NGS) technologies and computational tools such as mSINGS [5], MSISensor [6], and MANTIS [7] have enabled more comprehensive and precise interrogation of MSI across multiple cancer types. These tools demonstrate high sensitivity and specificity in detecting MSI, broadening the potential for MSI testing beyond the limitations of conventional methods. Emerging evidence suggests that MSI is a generalized feature across a broad spectrum of malignancies [8,9]. Furthermore, MSI has been recognized as an actionable biomarker for immune checkpoint blockade therapies, as patients with MSI-high (MSI-H) tumors respond favorably to inhibitors targeting the programmed cell death protein 1 (PD-1) pathway [10]. This response is likely due to enhanced recognition of neoantigens by T lymphocytes.

In canine cancers, MSI has been investigated in mammary gland tumors, with 63% of tumors exhibiting a significant MSI degree in 21 microsatellite markers in both blood and tumor tissues [11]. Another study evaluated MMR protein immunolabeling in eight different canine tumors using anti-human monoclonal antibodies [12]. A significant proportion of oral malignant melanomas and hepatocellular carcinomas showed reduced MMR activity, implying possible microsatellite instability. Additionally, a study of 101 dogs with various malignant tumors confirmed that MSI and deregulation of MMR (dMMR) were more prevalent in oral malignant melanomas, demonstrating a correlation between dMMR and MSI [13].

To gain a more comprehensive understanding of MSI prevalence across canine cancers, we utilized msiSensor-pro [14] to analyze MSI status in 10 distinct canine cancer histotypes, encompassing 692 tumors. This analysis was performed on whole-exome sequencing data, providing a genome-wide perspective on microsatellite instability. Our study aimed to elucidate the landscape of MSI across a wide array of malignancies and to explore its potential to identify tumors that could benefit from immunotherapy.

## 2. Materials and Methods

### 2.1. Sample Acquisition and Selection Criteria

Samples for this study were obtained from the Sequence Read Archive (SRA) database using the sra-toolkit command-line tool. Twenty studies, encompassing ten distinct canine cancer histotypes, were included in the analysis (Supplementary Table S1). Strict inclusion criteria were applied to ensure robust results. Only paired-end whole-exome sequencing data were considered, with each tumor sample requiring a matched healthy tissue sample from the same animal. Quality control measures were implemented to ensure adequate sequencing coverage.

The canine reference genome (canFam3.1) and corresponding gene annotation were obtained from the University of California, Santa Cruz (UCSC) Genome Browser website. These resources served as the foundation for subsequent analyses and variant calling. To facilitate the identification of somatic variants and minimize false positives, two databases containing known canine germline variants were utilized for filtering: the Dog Single Nucleotide Polymorphism Database (DogSD) [15] and the European Variation Archive (EVA) [16].

### 2.2. Data Preprocessing

The initial phase involved retrieving raw data and comprehensive study metadata, including accession numbers, tumor/normal status, and breed information, from each BioProject’s webpage. Python (version 3.9) and Bash scripts were used to organize cancer malignancies and match healthy sequencing data. The Burrows-Wheeler Aligner (BWA, version 0.7.17-r1188) [17] was employed to align each sample to the reference genome. Despite its somewhat outdated status, CanFam3.1 was chosen as the reference genome due to its widespread use in existing literature, ensuring maximum compatibility with available information. The resulting SAM files underwent processing using SAMtools (version 1.19.2) and Picard tools (version 2.27.5). Sequencing data were converted to BAM format, enriched with essential metadata, and sorted by coordinate. Finally, base quality score recalibration (BQSR) was performed using the BaseRecalibrator and ApplyBQSR tools from the Genome Analysis Toolkit (GATK, version 4.3.0.0) [18] to eliminate possible artifacts and correct quality scores.

### 2.3. Panel of Normals

To mitigate the impact of sequencing artifacts, a Panel of Normals (PON) was created for each analyzed BioProject, comprising mutations called in the corresponding healthy tissues. This approach, following GATK best practices, helped improve the accuracy of mutation identification by filtering out false positives and focusing on true somatic mutations.

### 2.4. Variant Calling

A majority voting approach was employed to identify single nucleotide variants (SNVs) and insertions/deletions (indels), using three callers: Mutect2 (version included in GATK 4.3.0.0) [19], Strelka (version 2.9.10) [20], and VarScan (version 2.4.3) [21]. Only aberrations retrieved from at least two out of three callers were considered trustworthy. This approach minimized the impact of caller-specific errors and biases. Annotations were performed using ANNOVAR (release 2020Jun08) [22].

### 2.5. Tumor Mutational Burden Analysis

Tumor mutational burden (TMB) was calculated to assess cancer genomic instability. The analysis quantified the total number of mutations per megabase of the coding genome sequenced, excluding mutations in mitochondrial DNA and unaligned chromosomes. The coding regions sequenced were limited to 57 Mb, estimated as the size of the canine exome target for each study.

### 2.6. Microsatellite Instability Analysis

MsiSensor-Pro [14] was used to perform MSI analysis. Candidate microsatellite regions of interest (ROI) were retrieved from the canFam3.1 reference genome. The tool assessed the number of repeats in the selected loci for all samples, comparing tumor and matched normal data simultaneously. Custom R scripts were used to select the most frequently affected ROI and to model statistical analysis, allowing for the identification of cancer-specific microsatellite instability patterns and their potential associations with other genomic features or clinical outcomes.

### 2.7. Statistical Analysis

All statistical analyses and data post-processing were performed in R (v4.3.2). The total number of candidate MSI loci was filtered to keep only the positions that were found in at least 5 samples. Then, the MSI burden was evaluated as the ratio between the number of filtered loci in each sample and the total number of relevant loci. Similarly, the tumor mutational burden (TMB) was calculated as the number of coding mutations found in each sample per megabase sequenced. A sample-wise comparison was first conducted between the MSI burden and TMB using Pearson correlation. Then, the samples were divided into three categories based on the MSI burden. If the burden was above the overall mean, the sample was categorized as MSI-H; if below, it was categorized as MSI-L; and if no loci were present, it was categorized as MSS. The TMB in all three categories was compared using the Wilcoxon test since the data were not normally distributed. All results were deemed statistically significant if *p*-value ≤ 0.05.

## 3. Results

We performed a comprehensive analysis of paired whole-exome sequencing data from 692 tumor-normal sample pairs, encompassing 10 distinct canine cancer subtypes. The sequencing data, obtained from previously published studies, comprised the following distribution: 137 melanomas, 136 mammary carcinomas, 103 B-cell lymphomas, 98 osteosarcomas, 65 T-cell lymphomas, 64 hemangiosarcomas, 52 gliomas, 28 mast cell tumors, 5 pulmonary adenocarcinomas, and 4 urinary carcinomas.

### 3.1. Microsatellite Instability

**Figure 1:**
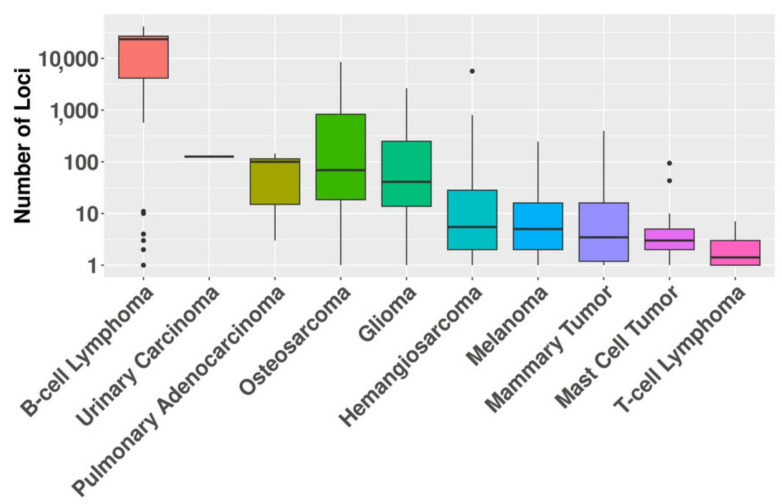
The graph displays the median number of affected loci on a logarithmic scale (*y*-axis) for each cancer histotype (*x*-axis).

MSI was detected in 446 (64%) tumors across the entire dataset. The prevalence of MSI varied substantially among cancer types, ranging from 100% in pulmonary adenocarcinomas to 29% in T-cell lymphomas. Analysis identified 1,019,895 repeated regions across the tumor sample cohort, distributed across 175,383 unique loci. The frequency of affected loci varied considerably, ranging from a single sample to 117 samples exhibiting alterations at a given locus. Significant differences (*p* < 0.001) in the mean number of microsatellite regions were observed among cancer histotypes, ranging from 2.3 in T-cell lymphomas to 19,209 in B-cell lymphomas (Figure 1, Supplementary Table S2). 

**Figure 2:**
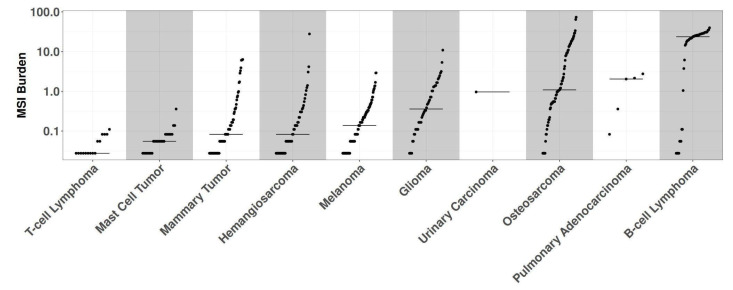
This figure displays the MSI burden (*y*-axis) for each sample by histotype (*x*-axis). T-cell lymphomas present the lowest burden, while B-cell lymphomas have the highest burden.

**Figure 3:**
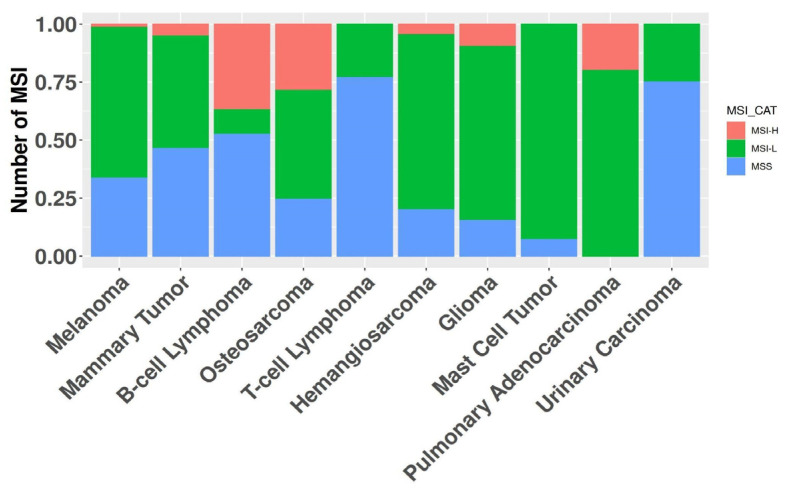
The graph depicts the number of samples (*y*-axis) for each cancer histotype (*x*-axis). The color-coded bars represent the proportion of tumors within each of the three microsatellite instability (MSI) classifications.

In the absence of an established consensus for selecting relevant genomic regions to study MSI in canine cancers, we implemented an arbitrary cutoff, retaining loci exhibiting alterations in at least five samples within the whole dataset. B-cell lymphomas were excluded from this analysis step due to their high number of MSI events, which could have obscured relevant loci in other cancer types. We retained 3632 regions for further analyses (Supplementary Table S3). Evaluation of the entire cohort using these newly identified loci revealed 430 samples harboring at least one MSI event, while 16 samples were excluded due to exhibiting only patient-specific MSI events. The median MSI burden varied substantially across histotypes, ranging from 19.2% in B-cell lymphomas to 0.05% in T-cell lymphomas (Figure 2). Modeling human MSI categorical classification, we classified tumor histotypes into three categories based on their MSI status: microsatellite stable (MSS) for samples without significant alterations in repeated regions; MSI high (MSI-H) for samples with an MSI burden above the mean value of 2.4%; and MSI low (MSI-L) for samples falling between these categories. Of the 10 cancer types exhibiting MSI, 7 had one or more MSI-H tumors present, with MSI-H prevalence ranging from 1.5% in melanomas to 37% in B-cell lymphomas (Figure 3). The relative level of instability varied considerably among MSI-H cancer types, spanning from 25.8% in B-cell lymphomas to 2.7% in pulmonary adenocarcinomas.

### 3.2. Correlation between Tumor Mutational Burden and MSI Status

**Figure 4:**
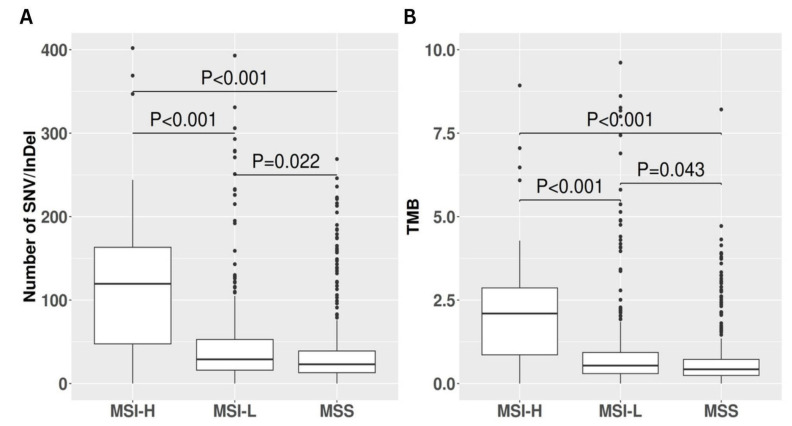
This panel illustrates two significant distinctions among microsatellite instability-high (MSI-H), microsatellite instability-low (MSI-L), and microsatellite stable (MSS) tumors, emphasizing the impact of high MSI levels on the mutational profile. (**A**) The graph demonstrates the substantial disparity in the number of affected somatic mutations across the three categories. (**B**) The right graph depicts the variation in TMB among MSI-H, MSI-L, and MSS.

**Figure 5:**
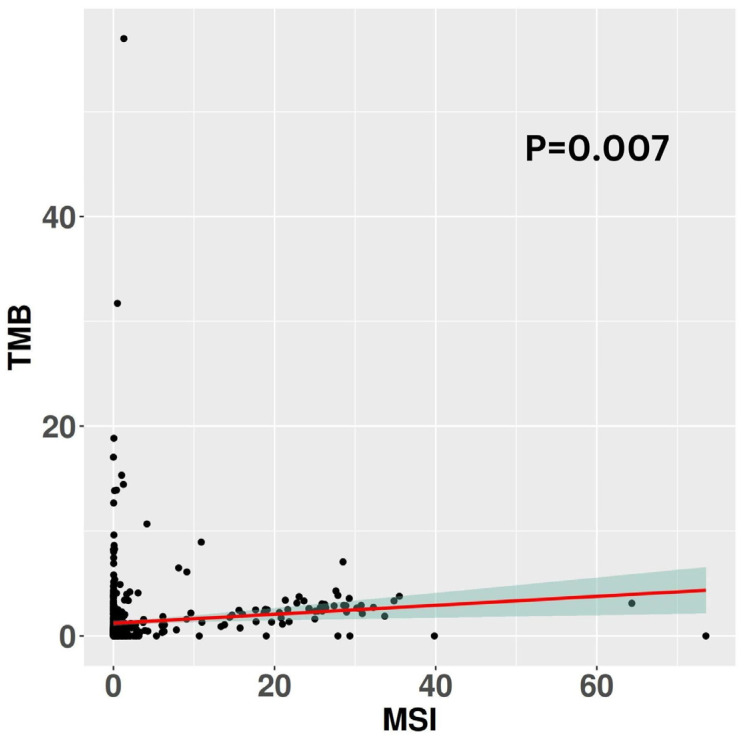
The image displays the per-sample correlation between TMB and MSI burden. The red line represents the fitted linear regression function derived from the data.

Analysis of somatic variants identified through whole-exome sequencing revealed that MSI-H tumors exhibited elevated average absolute numbers of both nonsynonymous and synonymous SNV and indels compared to MSS and MSI-L tumors. Indeed, MSI-H samples harbored a median of 119 (Q1: 47–Q3: 163) somatic mutations, significantly exceeding the median of 23 (Q1: 13–Q3: 39) somatic mutations observed in MSS samples (*p* < 0.001). MSI-L samples displayed an intermediate median of 29 (Q1: 16–Q3: 53) somatic mutations, significantly lower than MSI-H (*p* < 0.001) yet higher than MSS (*p* = 0.02); see Figure 4A. To further investigate the relationship between MSI and TMB, defined as the number of coding mutations per megabase sequenced, we assessed TMB across the MSI categories. MSI-H tumors exhibited a median TMB of 2.09 (Q1: 0.86–Q3: 2.86), while MSI-L and MSS tumors showed median TMB values of 0.54 (Q1: 0.29–Q3: 0.93) and 0.42 (Q1: 0.24–Q3: 0.72), respectively. MSI-H tumors demonstrated a significantly higher mutational burden compared to both MSI-L and MSS tumors (*p* < 0.001). Moreover, MSI-L tumors exhibited a significantly higher mutational burden than MSS tumors (*p* = 0.043). A visual representation is available in Figure 4B. Linear regression analysis comparing TMB with MSI burden revealed a weak yet statistically significant correlation between these variables (R = 0.03, *p* = 0.007); see Figure 5. 

## 4. Discussion

MSI is a well-established phenomenon in cancer biology, characterized by the accumulation of mutations in repetitive DNA sequences due to mismatch repair system defects. While extensively studied in human cancers, the prevalence and significance of MSI in canine cancers remain largely unexplored. This study aimed to address this knowledge gap by conducting a comprehensive analysis of MSI in canine cancer exomes, utilizing the largest dataset to date. Our investigation revealed a surprisingly high incidence of MSI, with 430 samples (63%) exhibiting instability. Although sample sizes for certain cancer types, such as pulmonary and urinary carcinomas, were relatively small, potentially affecting the reliability of prevalence estimates, the overall findings underscore the potential importance of MSI in canine cancer development and progression.

Notably, we observed significant heterogeneity in the extent and prevalence of MSI across different canine cancer histotypes. Some histotypes exhibited a high propensity for MSI events, while others displayed a relatively stable microsatellite landscape. Only three cancer types (mast cell tumors, T-cell lymphomas, and urinary carcinomas) did not present at least one MSI-high (MSI-H) sample. Conversely, B-cell lymphoma, osteosarcoma, glioma, mammary tumor, melanoma, hemangiosarcoma, and pulmonary carcinomas displayed higher levels of microsatellite instabilities. The heterogeneity observed across different canine cancer histotypes suggests that MSI may play varying roles in different tumor histologies, similar to what has been observed in human cancers [8]. In human oncology, MSI status has emerged as a powerful biomarker for predicting response to immune checkpoint inhibitors, particularly those targeting the PD-1/PD-L1 axis [10]. The high prevalence of MSI in canine cancers observed in our study suggests that this phenomenon could have significant implications for canine cancer immunotherapy as well. MSI-high tumors are characterized by an increased mutational burden, which can lead to the production of neoantigens that stimulate an anti-tumor immune response [23]. This increased immunogenicity makes MSI-high tumors particularly susceptible to immune checkpoint inhibition. Given the high prevalence of MSI in canine cancers, there may be a substantial subset of canine cancer patients who could benefit from immunotherapy approaches targeting PD-1 and PD-L1. Recent studies in canine oncology have begun to explore the potential of immune checkpoint inhibitors. For instance, Maekawa et al. (2017) demonstrated that canine PD-1 and PD-L1 function similarly to their human counterparts, suggesting that targeting this pathway could be effective in canine cancers [24]. Shosu et al. (2016) showed that PD-L1 expression in canine oral melanoma was associated with poor prognosis, mirroring findings in human melanoma [25]. Finally, drugs that involve PD-1/PD-L1 blockade, such as agents similar to Atezolizumab, have been tested. Additionally, others like Gilvetmab are now available on the market for treating mast cell tumors and melanoma [26,27]. 

The high prevalence of MSI in canine cancers, coupled with these emerging findings on immune checkpoint molecules, underscores the need for further investigation into the relationship between MSI status and response to immunotherapy in canine patients. Such studies could potentially lead to the development of MSI as a predictive biomarker for immunotherapy response in veterinary oncology, similar to its use in human medicine [28]. Moreover, the cancer-specific variations in MSI prevalence observed in our study suggest that certain canine cancer types may be more amenable to immunotherapy approaches than others. For instance, the high prevalence of MSI in canine B-cell lymphomas and hemangiosarcomas may indicate that these diseases could be particularly responsive to PD-1/PD-L1 blockade.

Notably, the number of MSI-affected loci in canine B-cell lymphomas was substantially higher than other canine cancers and previously reported in human studies, where this histotype typically exhibits a low overall MSI burden. This discrepancy may be attributed to the increased sequencing depth of B-cell lymphoma samples compared to other cancer types analyzed in this study. However, given the paucity of data on microsatellite instabilities in canine B-cell lymphomas, further investigation is warranted to elucidate the nature and significance of this phenomenon.

Our large sample size enabled the identification, with reasonable confidence, of the most recurrent genomic locations exhibiting microsatellite instability. We selected 3632 regions and successfully correlated them with the occurrence of single nucleotide variants and indels, affirming the significance of this aberration family in canine cancers. To classify tumors, we employed a score based on the ratio of MSI-affected loci to the total number of loci of interest. This measure was designed to parallel recent findings in human medicine, where studies have reported a strong correlation between the number of affected loci (and their ratios) and MSI status derived from laboratory techniques such as PCR [7]. We validated the relevance of this “MSI-burden” score by identifying a robust relationship with tumor mutational burden and the number of recurrently somatic mutations, thus revealing the biological implications of our newly defined metric.

Numerous studies in human medicine investigating microsatellite instabilities have employed MANTIS for MSI detection. However, we opted for msiSensor-pro, a more recent computational tool capable of detecting variants in both tumor-normal and tumor-only datasets. Previous performance comparisons between these tools have demonstrated superior results with msiSensor-pro. Moreover, the computation time for msiSensor-pro was significantly lower (approximately 3–4 min) compared to MANTIS (around 119 min), rendering it a more efficient choice for our analysis.

## 5. Conclusions

In conclusion, this pilot study presents the most comprehensive analysis of MSI in canine cancer exomes across 10 distinct histological subtypes to date. It is important to note that in our study, certain tumors were well represented, such as lymphoma, osteosarcoma, and melanoma. In contrast, other histotypes were underrepresented. Indeed, mast cell tumors, which have a high incidence in dogs, have been studied using WES in only a few cases. In humans, the majority of data on MSI have been obtained from colon cancer, but in dogs, this histotype is rare, and there is no publicly available WES data. The limited availability of WES data for some common canine tumors highlights the need for further genomic studies in veterinary oncology. Our findings reveal a surprisingly high overall incidence of MSI and significant heterogeneity across cancer types. These results underscore the potential importance of MSI in canine cancer biology, particularly in B-cell lymphomas, which exhibited the highest MSI burden and introduce a novel “MSI-burden” score, which correlates significantly with TMB and the number of recurrently mutated genes, providing a quantitative measure of MSI impact. These findings have limitations but highlight several key areas for further investigation, including the mechanistic relationship between MSI and canine cancer development, particularly in high-prevalence histotypes, the potential of MSI as a prognostic or predictive biomarker in canine oncology, and the correlation between MSI status and response to various treatment modalities, including traditional chemotherapies and targeted therapies. Finally, the high prevalence of MSI in canine cancers suggests a potential opportunity for immunotherapy approaches. Given the success of PD-1 and PD-L1 inhibitors in MSI-high human cancers, investigating these therapies in MSI-high canine cancers represents an exciting frontier in veterinary oncology.

## Data Availability

All data are available at https://github.com/eugeniomazzone/CanineMSI.git (accessed on 19 August 2024).

## Supplementary Materials

The following supporting information can be downloaded at: https://www.mdpi.com/article/10.3390/ani14172484/s1, Table S1: List of WES studies from which the data were collected [29,30,31,32,33,34,35,36,37,38,39,40,41,42,43,44,45,46,47,48]; Table S2: selected loci frequently affected by MSI with annotated genes; Table S3: MS and TMB description for each histotype.
